# Gut microbiota in obesity management: from microbial clocks to precision microbial therapies

**DOI:** 10.3389/fcimb.2026.1705021

**Published:** 2026-02-25

**Authors:** Liman Luo, Mei Xue, Li Sun, Zhe Dai

**Affiliations:** Division of Endocrinology and Department of Internal Medicine, Zhongnan Hospital of Wuhan University, Wuhan, China

**Keywords:** circadian dynamics, gut microbiota, microbial clocks, obesity, therapies

## Abstract

The gut microbiota exhibits robust circadian oscillations that synchronize with host metabolic cycles. Disruption of these microbial rhythms is increasingly recognized as a factor contributing to the pathogenesis of obesity. Clinical evidence supports that chrono-modulated interventions, including chrono-nutrition, temporal fecal microbiota transplantation (FMT), and engineered microbial systems, represent promising approaches in obesity management. This review synthesizes the features of gut microbiota circadian dynamics, the intrinsic and extrinsic factors regulating microbiota oscillations, and the precise microbial intervention measures targeting temporal patterns. Through the integration of insights into the microbiota-clock-metabolism axis, this review emphasizes the necessity of time-specific strategies in translating microbial circadian biology into effective, personalized obesity therapies.

## 1 Introduction

The global prevalence of obesity constitutes a significant public health challenge. Projections indicate that by 2030, 20% of the global population will be classified as obese. Associated healthcare costs are expected to reach 3 trillion US dollars annually (Okunogbe et al., 2022; NCD-RisC, 2024). This growing crisis is exacerbated by its metabolic comorbidities, including type 2 diabetes mellitus(T2DM), cardiovascular diseases, and non-alcoholic fatty liver disease (Abdelaal et al., 2017; Engin, 2017). Traditional weight management strategies, such as lifestyle modifications, pharmacotherapy, and bariatric surgery, often prove insufficient due to problems related to long-term adherence, adverse effects and surgical risks (Fink et al., 2022). These limitations highlight the urgent need for innovative approaches that address the fundamental causes of energy imbalance.

The gut microbiota acts as a key ecological determinant of obesity, influencing host metabolism through the extraction of dietary energy, the production of bioactive metabolites (e.g., short-chain fatty acids, secondary bile acids), and the interaction with immune and neuroendocrine systems. Recent advances underscore the gut microbiota and host’s intrinsic circadian rhythmicity (Xu et al., 2023; Min et al., 2024). Diurnal oscillations in microbial abundance and function synchronize with host circadian rhythms, thereby maintaining the host’s metabolic homeostasis. A defining feature of this microbial clock is the circadian rhythm of the *Firmicutes/Bacteroidetes* (F/B) ratio—the two dominant gut microbial phyla linked to obesity pathogenesis. Notably, impaired microbial rhythmicity in obesity is characterized not merely by a shifted F/B ratio, but by a dampened amplitude of its circadian oscillations, alongside reduced gut microbiome diversity and richness (Vallianou et al., 2019; Geng et al., 2022; Gradisteanu et al., 2022). This distinct rhythmic disruption, rather than simple compositional imbalance, is emerging as a key driver of metabolic dysregulation.

However, current probiotic and dietary interventions usually fail to utilize time specificity. The mechanistic links between microbial clocks and host metabolic regulation remain poorly defined, which to some extent impedes the translation of microbial clocks into precision obesity therapies. This review explores the microbiota-clock-metabolism axis, where disrupted microbial oscillations drive metabolic dysregulation. We evaluate evidence linking microbial chrono-disruption to obesity and examine innovative microbial therapeutic approaches for resetting host-microbial rhythms.

## 2 Circadian regulation of gut microbiota and host metabolism

### 2.1 Diurnal rhythm characteristics of the gut microbiota

The gut microbiota is a dynamic ecosystem shaped by host circadian rhythms and dietary cycles. Its composition and function exhibit hourly fluctuations, following a 24-hour rhythm. This generates robust diurnal oscillations and time-of-day-specific profiles over the course of a day (Thaiss et al., 2014; Kiessling et al., 2025).

Up to 60% of rodent gut bacteria display circadian rhythms in relative and absolute abundance (Heddes et al., 2022). And the periodic shifts in the gut microbiome are evident in all major phyla (Zarrinpar et al., 2014). In particular, the two dominant phyla of the gut microbiome, *Firmicutes* and *Bacteroides*, display the most pronounced diurnal fluctuations. In humans, the relative abundance of *Bacteroidetes* at night is approximately 6% higher than during the day, whereas the abundance of *Firmicutes* is elevated in the daytime (Reitmeier et al., 2020). In mice, the *Firmicutes* phylum peaks at the end of the feeding period, while *Bacteroidetes* has a significant peak during the fasting period. Both phyla exhibit stable diurnal alternating fluctuations (Zarrinpar et al., 2014; Reitmeier et al., 2020; Litichevskiy and Thaiss, 2022). Liang et al. (2015) further reported that the overall rhythmicity of the gut microbiota is driven by *Bacteroidetes*, whereas *Firmicutes* maintains relatively constant absolute abundance.

Additionally, approximately 10%-20% of human microbial taxa exhibit diurnal oscillations, including the genera *Parabacteroides*, *Lachnospira*, and *Bulleidia (*Reitmeier et al., 2020). The individual variations in microbiota structure potentially influenced by defecation timing. Interestingly, the gut microbiota also modulates the rhythmic patterns of microbial metabolite production. As the primary microbial metabolites, short-chain fatty acids (SCFAs) and secondary bile acids exhibit circadian oscillations in the intestine and serum (Segers et al., 2019; Wang et al., 2020; Allaband et al., 2021).The research have reported that cecal contents from mice fed a regular chow diet display a distinct, diurnal pattern of SCFAs concentration (Leone et al., 2015).

### 2.2 Disrupted rhythmicity of gut microbiota in obesity

Circadian desynchronization of the gut microbiota is closely associated with obesity, T2DM and related metabolic disorders (Reitmeier et al., 2020; Altaha et al., 2022).Studies have demonstrated that obese humans and mice have different intestinal microbiota from the lean controls. Transfer of microbiota from obese humans or mice to lean mice undergoing jetlag induces obesity-related phenotypes (Thaiss et al., 2014; Zecheng et al., 2023).The core rhythmic imbalance of the F/B ratio is a hallmark of the obese state. Even some studies report no significant shift in the absolute F/B ratio, the amplitude of its circadian oscillations is reduced by 30% to 50% in obese individuals (Reitmeier et al., 2020). In obese mice, the rhythmic dominance of the Firmicutes phylum was enhanced, and the amplitude of the Bacteroidetes phylum rhythm decreased by approximately 40% compared with that in normal mice, with the peak disappearing during the fasting period. This F/B rhythmic dysregulation is directly associated with obesity-related metabolic abnormalities (Zarrinpar et al., 2014; Yin et al., 2020). Furthermore, metabolic disorders related to obesity can lead to a significant decrease in the proportion of periodic OTUs (operational taxonomic units) in the gut microbiota (Yin et al., 2020). Of note, the metabolic derivatives of the gut microbiota in obese individuals also show different circadian rhythm changes. The metabolomics determination results show that the circadian rhythms of bile acids in the serum, cecal contents, intestine and liver of ob/ob mice were disrupted compared with those of ob/m mice (Guo et al., 2024). The obese mice lose circadian coordination between microbial bile acid metabolism and hepatic FXR signaling (Zhao et al., 2025).

These observations confirm that circadian oscillations in the gut microbiota and metabolites is closely related to obesity. Humans and obese mice both exhibit an imbalance in the F/B ratio rhythm and a decrease in the alpha diversity of the microbiota. The difference is that the disappearance of the rhythm of Verrucomicrobia (such as the genus Akkermansia) in mice is a typical feature of obesity (Zarrinpar et al., 2014; Reitmeier et al., 2020), while the rhythm changes of this phylum in obese humans have not yet reached a unified conclusion. Some studies have only observed a reduction in its abundance rather than rhythmic disorder (Yin et al., 2020).

### 2.3 Rhythmic regulation of the gut microbiota

The circadian rhythm of host, regulated by gene-encoded molecular clocks, plays a pivotal role in coordinating the rhythmic fluctuations in both the composition and function of the gut microbiota (Takahashi, 2017). Microbial oscillations are not directly entrained by light-dark cycles but are instead affected by the host’s circadian adaptive mechanisms, including the light-dark cycles, feeding behavior and sleep-wake patterns (Takahashi, 2017; Kiessling et al., 2025). As observed in mice, the fluctuations of *Bacteroidetes* exhibits particularly pronounced diurnal fluctuations. However, there was the disruption of microbiota rhythmicity in the Bmal1-deficient mice (Liang et al., 2015; Altaha et al., 2022), and a loss of microbial richness and diversity in ClockΔ19 individuals (Parkar et al., 2019). Knockout of the biological clock gene Bmal1 alters the diurnal fluctuations of *Bacteroidetes*, *Firmicutes*, and *Proteobacteria*, while concurrently increasing the abundance of the genus *Rikenella (*Liang et al., 2015). Moreover, environmental light cycles entrain circadian feeding behaviors in animals, which subsequently generates rhythms in exposure to food-borne bacteria (Brooks et al., 2021). For instance, mice with scheduled eating exhibit cyclical fluctuations in 17% of detected operational taxonomic units(OTUs) (Zarrinpar et al., 2014).

Dietary changes within a 24-hour period rapidly alter gut microbiome composition (Ross et al., 2024). Misaligned meal timing, such as skipping breakfast, overeating at night, or ad libitum feeding without defined fasting-feeding cycles, disrupted the circadian rhythm and the gut microbiota clock, inducing the occurrence of metabolic disorders such as obesity and T2DM (Jakubowicz et al., 2024).Therefore, dietary composition and rhythms critically modulate the microbiota-clock axis. Specifically, HFD disrupts specific host factors (e.g., Reg3γ) and microbial circadian rhythms associated with the circadian pattern of microorganisms, which in turn promotes metabolic dysfunction (Frazier et al., 2022). Conversely, time-restricted feeding (TRF), an 8-hour feeding window in obese mice, restores periodic oscillations in microbial community structure and mitigates metabolic dysfunction, including rescuing the oscillations of the F/B ratio (Ye et al., 2020)., Microbial functions differ markedly between satiation and starvation conditions. The abundance of protective genera such as *Lactobacillus*, *Oscillibacter*, *Romboutsia* and *Lachnoclostridium* peaks in the feeding phase, while the fattening genera such as *Faecalibaculum*, *Lactococcus* and *Streptococcus* peaked in the starvation phase. Notably, the bacterial communities that colonize the gut during the feeding phase exert distinct beneficial metabolic effects, unlike those present during fasting (Xia et al., 2023).However, Reitmeier et al. proposed that dietary habits and dietary intake may not be represent the primary drivers of the abnormal microbiota composition (Reitmeier et al., 2020).

Rhythmic feeding is critical for maintaining the homeostasis of microbial community structure and sustaining diurnal fluctuations in microbial abundance (Brooks et al., 2021).Recent studies have shown that dietary-timing induces diurnal oscillations of *P. distasonis*, which in turn modulates the host’s inflammatory rhythms (Ma et al., 2024). Importantly, the regulation of microbial rhythms by eating times is closely linked to host circadian rhythm genes. TRF exerts a more pronounced effect on restoring the amplitude of microbial rhythms when host clock genes(e.g., Clock) function normally. Conversely, even regular feeding patterns fails to effectively restore the rhythmic fluctuations of key microbial taxa in hosts with impaired clock gene function (Parkar et al., 2019; Lotti et al., 2023). These findings indicate that host intrinsic circadian genes form the molecular basis for microbial rhythm regulation, with dietary timing acting as a key a key modifiable external factor. Disordered meal timing additionally leads to asynchronous circadian rhythms and gut microbiota oscillations, which is associated with insulin resistance and abnormal glycolipid metabolism (Gutierrez et al., 2021; Lotti et al., 2023; Jakubowicz et al., 2024).In contrast, a balanced, time-structured diet preserves intestinal circadian rhythm homeostasis, especially by regulating the rhythm fluctuations of specific obesity-related bacteria. Importantly, the first meal after a long fast, most commonly breakfast, resets the phase of the peripheral clock (He et al., 2021).This resetting effect is mediated by the rhythmic exposure of gut microbiota to dietary components, thereby enhancing the synchronization between microbial oscillations and host metabolic cycles. For example, morning intake of prebiotics or *Helianthus tuberosus* modulates *Bacteroidetes* more effectively than evening intake, which may be attributed to the phase-resetting role of breakfast in the peripheral clock (Sasaki et al., 2019; Niu et al., 2024).

Collectively, the host circadian genes, feeding rhythms, and dietary components constitute a regulatory network for gut microbiota rhythms. Among these factors, feeding timing emerges as a core modifiable target, as it directly modulates microbial oscillations by resetting the peripheral clock. This regulatory mechanism not only explains the link between disordered eating patterns and microbial rhythm disruption but also provides a mechanistic basis for innovative obesity interventions including chrono-nutrition and TRF.

## 3 Precision microbiota-targeted interventions

### 3.1 Timed administration of probiotics and prebiotics

Probiotic or prebiotic supplements targeting gut microbiota are emerging as a promising approach to comprehensive nutritional intervention for managing obesity. With the increasing emphasis on microbial clocks, the timing of administration has gradually drawn attention. However, there is no clear definition of the time to take probiotics or prebiotics.

The survival rate of probiotics at different administration times has been investigated through an *in vitro* digestion model. Probiotic survival rate was the highest when administered with a meal or 30 minutes preprandially, multi-strain probiotics containing *Lactobacillus helveticus R0052 and Lactobacillus rhamnosus R0011*, *Bifidobacterium longum R0175* and *Saccharomyces cerevisiae boulardii* exhibit the highest intestinal survival rates (Tompkins et al., 2011).Conversely, Wang J et al. believe that co-ingestion with food may buffer gastrointestinal stress and enhance the survival rate of probiotics. The survival rate of probiotics taken with or after meals is higher than that on an empty stomach (Wang et al., 2025).In humans, taking multi-strain probiotic capsules after meals reduced the BMI of overweight/obese adults by approximately 2.50% (Sudha et al., 2019). Further, probiotic of *Bifidobacterium animalis lactis BL-99* taken with each of three meals and at 21:00 before going to sleep ameliorated lipid metabolism through elevating short-chain fatty acids (Li et al., 2023).A clinical study has shown that taking Lactobacillus reuteri in the evening can improve blood sugar control and sleep quality in people with circadian rhythm disorders (Abildgaard et al., 2017).The differences in these results may be the interaction between probiotic cells and the surrounding food matrix. Currently, there are few clinical trials describing the specific administration methods of probiotics. Overall, ensuring the alignment of probiotic activity with the host’s circadian rhythm can optimize the therapeutic efficacy of probiotics. In the future, more *in-vitro* and clinical studies are required to clarify these methods.

In addition, the supplementation of prebiotics exerts time-dependent differences in therapeutic outcomes. The animal experiment results indicate that supplementing inulin at night is more beneficial than in the morning (Chen et al., 2025).Supplementation of sodium butyrate in the morning during the peak period of SCFAs receptor activity in the circadian rhythm is more effective than at non-rhythmically matched times (Li et al., 2025).However, mice and humans exhibit different circadian rhythms, which leads to differences in human results compared to animal experiments. For instance, inulin intake in the morning rather than the evening strongly impacted on the composition of the microbiota (Sasaki et al., 2019).The relative abundance of *Bacteroidetes* was significantly higher and *Firmicutes* was significantly lower in the morning inulin group than in the evening group (Sasaki et al., 2019).Therefore, this is also more conducive to improving the loss of rhythm of *Bacteroides* and *Firmicutes* in obese individuals. Moreover, taking *Helianthus tuberosus* at breakfast rather than at dinner is more effective for improving the intestinal environment and intestinal microbiota (Kim et al., 2020).The first meal after a long fast, most often breakfast, resets the phase of the peripheral clock. And, appropriate administration time enhances the effectiveness of the intervention. Taking prebiotics at different times may lead to different microbial environments and host physiological states, thereby resulting in differences in intervention effects.

### 3.2 Chrono-nutrition: synchronizing microbial clocks

Diet patterns and meal timing as a determinant of microbial rhythmicity, reduced the risk of underlying diseases by synchronizing food intake with the circadian rhythm cycle. It is reported that eating at the appropriate time in biology, especially earlier in the day when the circadian metabolic is at its peak, greatly improved the intestinal microbiota rhythm and promoted the production of SCFAs. Dietary-timing-induced gut microbiota diurnal oscillations modulate inflammatory rhythm, especially the fluctuation of *P.distasonis (*Ma et al., 2024).Therefore, it is crucial to pay attention to the eating window for targeted management of the gut microbiota.

Intermittent fasting(IF), including periodic fasting and TRF, has been increasingly suggested to alleviate obesity, steatohepatitis and other conditions by resynchronizing the rhythmic oscillations of the gut microbiota (Xia et al., 2023; Li et al., 2024; Niu et al., 2024). In HFD-induced obese mice, an 8-hour TRF regimen restored the diurnal oscillations of *Bacteroidetes* and *Firmicutes*, whose rhythmic imbalance is a hallmark of obesity (Ye et al., 2020). Metagenomic analysis further revealed that during the feeding stage, TRF enriched the probiotic species involved in the synthesis of short-chain fatty acids, such as *lactic acid bacteria* and *Oscillibacter*. The fasting stage is associated with an increase in the abundance of potential fattening bacteria, such as *Faecalibaculum* and *Streptococcus (*Xia et al., 2023).Oolong tea polyphenols, as a natural antioxidant, improved the circadian rhythm disorder of specific gut microbiota in mice and alleviated the disorder of hepatic clock gene expression induced by constant darkness (Guo et al., 2019).Similarly, a methionine-restricted diet partially restored the arrhythmicity of the gut microbiota caused by a high-fat diet in mice (Wang et al., 2020).This experimental evidence collectively demonstrate that TRF and targeted dietary interventions have the potential to reset microbial clocks. However, the translational significance of this findings to humans remain to be validated due to interspecies differences in circadian physiology. Early clinical studies have explored the feasibility of chrono-nutrition among overweight or obese populations. For instance, the 12-week restricted feeding with an average duration of 10.47 hours from the start to the end of each day reduces the average weight of overweight or obese individuals by about 5% (Li et al., 2024). Metagenomic analysis of the microflora showed that TRF intervention partially restores these cyclical fluctuations, and increased the abundance of probiotic species *Parabacteroides distasonis*, *Bacteroides intestinalis*, *Parabacteroides goldsteinii*, and *Escherichia coli* in obese individuals (Li et al., 2024). Furthermore, 8-hours TRF achieved a greater reduction in BMI and soft lean body mass loss by enriching the abundance of probiotic species involved in the synthesis of SCFAs, and elevated fecal DCA and IAA concentrations compared with eating throughout the day (Li et al., 2024). Notably, only the microbiota derived from the TRF feeding phase, rather than that derived from the TRF fasting phase, restored the rhythm of the microbiota, confirming that the microbiota improves obesity in a specific manner at a times of the day (Xia et al., 2023). Large-scale randomized controlled trials further demonstrated the great potential of timed nutrition in obesity management. A meta-analyses of 11 RCTs in overweight or obese patients displayed that intermittent energy restriction improved weight loss and reduced body fat versus continuous energy restriction (He et al., 2021; Patikorn et al., 2021).

Therefore, early TRF, which restricts food consumption to the regular window of 8 to 10 hours each day, is one of the most promising non-pharmaceutical treatment methods for management of obesity. Future research in chrono-nutrition should focus on developing personalized meal times and dietary strategies to adapt to individual chronotypes and the characteristics of their gut microbiota.

### 3.3 Fecal microbiota transplantation

Fecal microbiota transplantation (FMT), which restored the function and structure of the gut microbiota by transferring specific microbial communities from healthy donors through capsules or endoscopy, has emerged as a potential therapeutic strategy for obesity (Khoruts and Sadowsky, 2016; Allegretti et al., 2020; Mocanu et al., 2021; Ng et al., 2022; Zhang et al., 2024). FMT reconstructs the homeostasis of the gut microbiota and exerts beneficial effects by introducing a variety of beneficial bacteria to patients (Kootte et al., 2017; Leong et al., 2020). Preclinical studies have provided foundational insights into FMT’s metabolic effects. For instance, FMT regulated glucose and lipid metabolism of mice by enhancing the abundance of anti-obesity bacterial strains such as *Akkermansia*, *Bacteroides* and *Blautia (*Wang et al., 2025).Notably, the temporal dynamic characteristics of donor microorganisms may influence the therapeutic efficacy. Morning FMT was more effective in improving insulin sensitivity and body weight than evening FMT (Zarrinpar et al., 2014).Additionally, germ-free mice receiving FMT showed that the colonization process of the microbiota was controlled by the host’s biological clock, emphasizing the importance of timing in FMT protocols (Liang et al., 2015). In a chronic unpredictable mild stress mouse model, an appropriate FMT time window enhanced the colonization and functional exertion of beneficial bacteria (Cao et al., 2026).This further supports that the therapeutic effect of FMT is dependent on the circadian rhythm.

Currently, preliminary clinical investigations have explored the feasibility of FMT in obese populations. Randomized controlled trials have provided more robust evidence for the effect of FMT. A small-scale study reported that repeated FMT administrations or combination with lifestyle interventions enhanced the level and duration of microbiota implantation in obese patients, which was associated with improvements in lipid profiles and liver stiffness (Ng et al., 2022).Large-scale RCTs have provided more robust evidence for FMT’s effects. An RCT demonstrated that FMT from lean donors reduces the proportion of abdominal fat in obese adolescents and improves their insulin sensitivity (Leong et al., 2020). Meanwhile, the enrichment effect of FMT on *Paraprevotella*, *Longibaculum* and *C. hylemonae* may have contributed to enhancing the intestinal microbiota bile acid metabolism and/or slow the development of glucose intolerance in obese patients (Bustamante et al., 2022).Meta-analyses of early RCTs also suggest that FMT may modulate key metabolic pathways, though consistent effects on BMI remains to be established (Kootte et al., 2017; Allegretti et al., 2020; Yu et al., 2020; Rinott et al., 2021).

Nevertheless, due to the complexity of microbial composition and individual differences, there are fluctuations in their efficacy and stability. The donor selection, individual patient variations, and rhythmic stability lead to the implantation and therapeutic effects of FMT. Healthy donors with higher microbial diversity and stable microbiota rhythms enhance the colonization ability of the recipient’s gut microbiota improve the efficacy of FMT (Buffie et al., 2015; Li et al., 2017; Hou et al., 2025).However, the degree of donor strain engraftment varied substantially between FMT recipients (Wilson et al., 2021).Studies shown that oral capsule microbiota transplantation has delayed implantation, despite the clinical efficacy is similar compared with the endoscopic FMT. Within 1 week following colonoscopy microbiota transplantation, the patient’s microbiota was similar to normalization (Jalanka et al., 2016), while engraftment levels of oral capsule transplantation similar to that of colonoscopy until 2–4 weeks following administration (Jiang et al., 2018). Additionally, as observed in animal studies, FMT time may modulate therapeutic efficacy, but this factor has not been systematically evaluated in human trials (Zarrinpar et al., 2014; Yu et al., 2020).The effects of FMT on body weight vary with concurrent dietary interventions. For example, the effects of autologous FMT show greater weight loss benefits when combined with calorie restriction compared to FMT alone (Rinott et al., 2021). Conversely, some trials report no significant improvement in BMI or metabolic parameters after FMT. This may be due to inadequate adjunct support or poor donor-recipient matching (Allegretti et al., 2020; Leong et al., 2020; Yu et al., 2020).

In summary, FMT shows potential for the management of obesity, especially when customized according to the circadian rhythms of donors and recipients and integrated with lifestyle interventions. Future research should focus on optimizing the selection of donors, considering the “chrono-FMT” approaches, which maximize metabolic benefits in obesity treatment by matching the donor’s microbial rhythm with the recipient’s circadian cycle.

### 3.4 Personalized biotherapy: engineered probiotics

Advances in genetic engineering technologies has enabled probiotics to exhibit targeted therapeutic properties. Targeted modification of strains may enhance the therapeutic efficacy in disease treatment, including obesity. For example, modified *Escherichia coli Nissle 1917 (E coli.N 1917)* that exerted great potential in obesity and cardiometabolic diseases secrete dipeptidyl peptidase 4-degradation-resistant glucagon-like peptide(GLP-1) or N-acyl phosphatidylethanolamine (Ma et al., 2020).Administration of *E coli.N 1917* for 8 weeks significantly decreased body weight, body weight gain, food intake, fat pad weight, and hepatic weight of HFD mice (Ma et al., 2020).Similarly, engineered *Lactobacillus paracasei F19* enables localized therapeutic delivery. Strains modified to overexpress N-acylphosphatidylethanolamine (NAPE) directly in the colon demonstrated significant anti-obesity effects, including decreased appetite, reduced body weight gain, and improved glucose metabolism and fat mass deposition (Seguella et al., 2025; Shen et al., 2025). And the research observed enrichment of *Prevotella* and *Parabacteroides* and modulation of the microbiota biodiversity in mice treated with oleoylethanolamide-producing *Lactobacillus paracasei F19 (*Seguella et al., 2025).The administration of *Lactobacillus paracasei F19* expressing NAPE and oleate elicited a sustainable decrease in body weight of mice on a high-fat diet and markedly improved metabolic syndrome by significantly reducing energy intake (Seguella et al., 2025).However, no such significant effects were observed in obese mice treated with the native probiotic alone (Seguella et al., 2025). Additionally, the modified *Lactobacillus* improved the metabolic dysfunction induced by a high-fat diet by secreting glucagon-like peptide (Ryan et al., 2017).Engineered probiotic *Escherichia coli* targets indoleacetic acid production and detect specific inflammation markers like thiosulfate and nitrate to deploy therapy (Woo et al., 2025).A great deal of experimental evidence shows that engineered probiotics perform significantly better than traditional probiotics in terms of colonization ability, pathogen rejection, barrier protection and immune regulation.

However, despite the encouraging results of preclinical studies, there is still a risk of translational failure in later clinical trials. The development of engineered beneficial bacteria requires comprehensive consideration of multiple aspects, such as the selection of chassis strains and the application of genetic engineering technology. At the same time, the clinical application of engineered probiotics still faces major challenges in terms of biosafety, regulatory compliance and environmental ethics. For instance, the long-term colonization of engineered bacteria may lead to their entry into the external environment, posing ecological risks.

## 4 Conclusion

The gut microbiota is a dynamic ecosystem that interacts with the host’s integrated circadian physiological network. The robust diurnal oscillations in microbial composition and metabolite production form the basis of metabolic homeostasis. Critically, obesity is driven not by simple gut microbial compositional imbalance, but by circadian desynchronization of the microbiota-clock-metabolism axis. This rhythmic disruption, accompanied by reduced microbial diversity and disordered metabolite oscillations, represents a core ecological determinant of obesity and its metabolic comorbidities that has been underappreciated in traditional weight management strategies.

**Figure 1:**
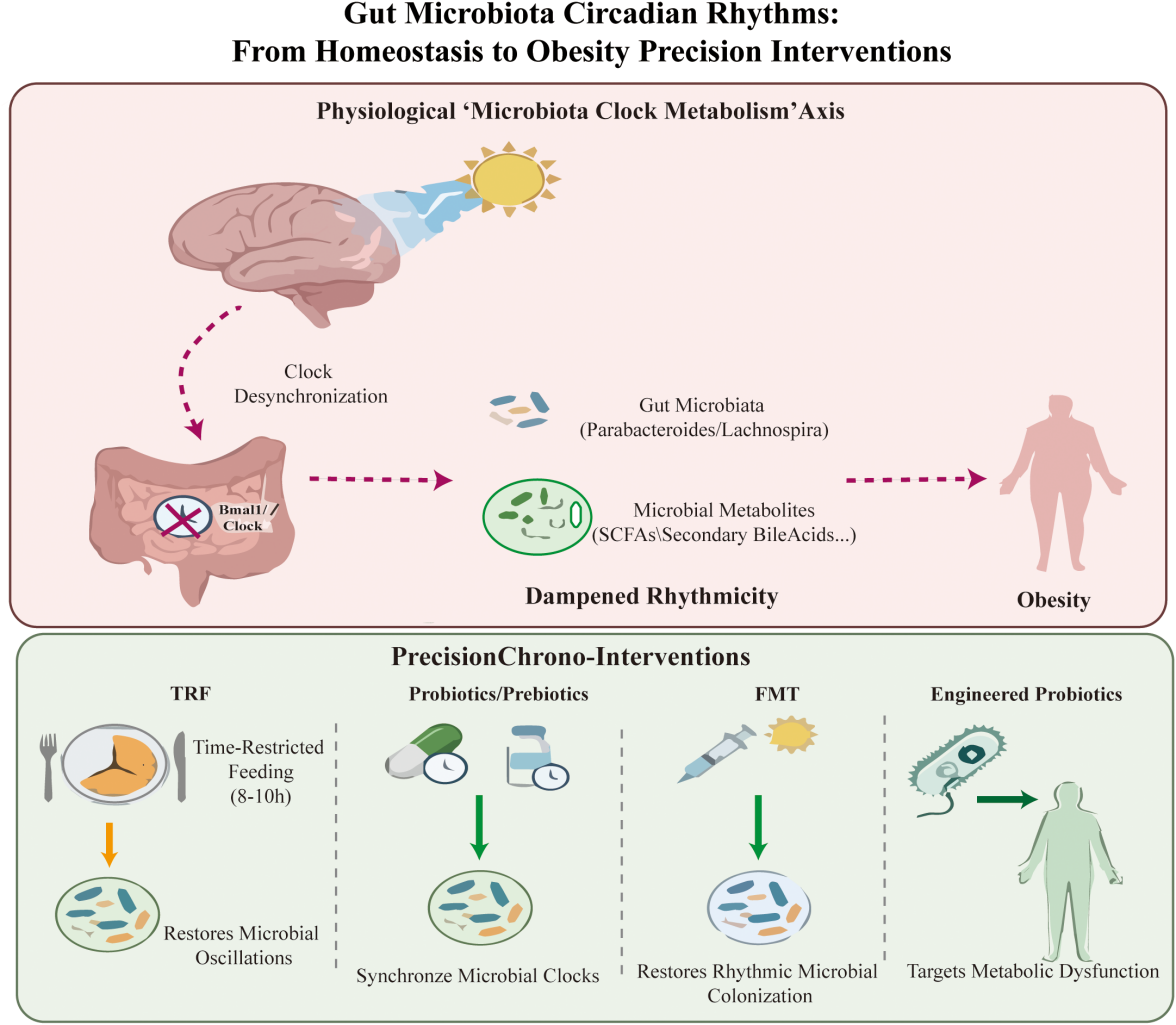
The microbial-clock-metabolism axis and the precision intervention strategies in obesity. This figure illustrates the key dynamics of the microbiota-clock-metabolism axis in obesity pathogenesis and targeted intervention. Host core clock genes (e.g., Bmal1) regulate circadian oscillations of key gut microbiota taxa and microbial metabolites synchronize with the host’s metabolic cycle to maintain metabolic balance. Circadian desynchronization of this axis causes dampened microbial rhythmicity and disrupted metabolite oscillations, which drives the development of obesity. Precision chronotherapeutic strategies (time-restricted feeding, timed probiotic/prebiotic administration, fecal microbiota transplantation, engineered probiotics) restore microbiota circadian oscillations and e-synchronize the microbiota-clock-metabolism axis, thereby alleviating obesity-related metabolic dysfunction.

This review systematically integrates and advances the field by constructing a time-centric precision microbial intervention framework targeting the microbiota-clock-metabolism axis for obesity management. We comprehensively summarize the therapeutic potential of time-specific interventions, including timed probiotic/prebiotic administration, chrono-nutrition, chrono-FMT, and engineered probiotics, all of which act by resynchronizing microbial circadian oscillations (**Figure 1**).

Despite the promising evidence for clock-targeted microbial therapies, significant gaps remain in this field. First, the causality in microbiota rhythms and obesity requires validation in large-scale longitudinal human studies. Second, the molecular mechanisms underlying the crosstalk between host circadian genes and microbial rhythmicity remain incompletely elucidated, particularly the downstream signaling pathways mediating microbial clock regulation of host metabolism. Third, the biosafety and ethical implications of engineered microbes demand rigorous preclinical and clinical evaluation. The precise therapy of gut microbiota and microbiota rhythm in obesity-related metabolic diseases still needs further exploration.

## References

[B1] Abdelaal M. le Roux C. W. Docherty N. G. (2017). Morbidity and mortality associated with obesity. Ann. Transl. Med. 5, 161. doi: 10.21037/atm.2017.03.107, PMID: 28480197 PMC5401682

[B2] Abildgaard A. Elfving B. Hokland M. Wegener G. Lund S. (2017). Probiotic treatment reduces depressive-like behaviour in rats independently of diet. Psychoneuroendocrinology 79, 40–48. doi: 10.1016/j.psyneuen.2017.02.014, PMID: 28259042

[B3] Allaband C. Lingaraju A. Martino C. Russell B. Tripathi A. Poulsen O. . (2021). Intermittent hypoxia and hypercapnia alter diurnal rhythms of luminal gut microbiome and metabolome. mSystems 6, e0011621. doi: 10.1128/mSystems.00116-21, PMID: 34184915 PMC8269208

[B4] Allegretti J. R. . (2020). Effects of fecal microbiota transplantation with oral capsules in obese patients. Clin. Gastroenterol. Hepatol. 18, 855–863.e2. doi: 10.1016/j.cgh.2019.07.006, PMID: 31301451

[B5] Altaha B. Heddes M. Pilorz V. Niu Y. Gorbunova E. Gigl M. . (2022). Genetic and environmental circadian disruption induce weight gain through changes in the gut microbiome. Mol. Metab. 66, 101628. doi: 10.1016/j.molmet.2022.101628, PMID: 36334897 PMC9672454

[B6] Brooks J. F. II Behrendt C. L. Ruhn K. A. Lee S. Raj P. Takahashi J. S. . (2021). The microbiota coordinates diurnal rhythms in innate immunity with the circadian clock. Cell 184, 4154–4167.e12. doi: 10.1016/j.cell.2021.07.001, PMID: 34324837 PMC8967342

[B7] Buffie C. G. Bucci V. Stein R. R. McKenney P. T. Ling L. Gobourne A. . (2015). Precision microbiome reconstitution restores bile acid mediated resistance to Clostridium difficile. Nature 517, 205–208. doi: 10.1038/nature13828, PMID: 25337874 PMC4354891

[B8] Bustamante J. M. Dawson T. Loeffler C. Marfori Z. Marchesi J. R. Mullish B. H. . (2022). Impact of fecal microbiota transplantation on gut bacterial bile acid metabolism in humans. Nutrients 14. doi: 10.3390/nu14245200, PMID: 36558359 PMC9785599

[B9] Cao P. Li Y. Zhang S. Li C. Sun Y. An B. (2026). Study on the efficacy and mechanism of fecal microbiota transplantation for depression based on circadian rhythm. Brain Behavior Immun. 131, 106186. doi: 10.1016/j.bbi.2025.106186, PMID: 41265661

[B10] Chen P. Chen F. Hou T. Hu X. Xia C. Zhang J. . (2025). Administration time modify the anxiolytic and antidepressant effects of inulin via gut-brain axis. Int. J. Biol. Macromol 288, 138698. doi: 10.1016/j.ijbiomac.2024.138698, PMID: 39672439

[B11] Engin A. (2017). The definition and prevalence of obesity and metabolic syndrome. Adv. Exp. Med. Biol. 960, 1–17. doi: 10.1007/978-3-319-48382-5 28585193

[B12] Fink J. Seifert G. Blüher M. Fichtner-Feigl S. Marjanovic G. (2022). Obesity surgery. Dtsch Arztebl Int. 119, 70–80. doi: 10.3238/arztebl.m2021.0359, PMID: 34819222 PMC9059860

[B13] Frazier K. Kambal A. Zale E. A. Pierre J. F. Hubert N. Miyoshi S. . (2022). High-fat diet disrupts REG3gamma and gut microbial rhythms promoting metabolic dysfunction. Cell Host Microbe 30, 809–823.e6. doi: 10.1016/j.chom.2022.03.030, PMID: 35439436 PMC9281554

[B14] Geng J. Ni Q. Sun W. Li L. Feng X. (2022). The links between gut microbiota and obesity and obesity related diseases. BioMed. Pharmacother. 147, 112678. doi: 10.1016/j.biopha.2022.112678, PMID: 35134709

[B15] Gradisteanu Pircalabioru G. Ilie I. Oprea L. Picu A. Petcu L. M. Burlibasa L. . (2022). Microbiome, mycobiome and related metabolites alterations in patients with metabolic syndrome-A pilot study. Metabolites 12, 218. doi: 10.3390/metabo12030218, PMID: 35323661 PMC8951583

[B16] Guo T. Ho C. T. Zhang X. Cao J. Wang H. Shao X. . (2019). Oolong tea polyphenols ameliorate circadian rhythm of intestinal microbiome and liver clock genes in mouse model. J. Agric. Food Chem. 67, 11969–11976. doi: 10.1021/acs.jafc.9b04869, PMID: 31583884

[B17] Guo X. Wang J. Xu H. Wang Y. Cao Y. Wen Y. . (2024). Obesity induced disruption on diurnal rhythm of insulin sensitivity via gut microbiome-bile acid metabolism. Biochim. Biophys. Acta Mol. Cell Biol. Lipids 1869, 159419. doi: 10.1016/j.bbalip.2023.159419, PMID: 37951383

[B18] Gutierrez Lopez D. E. Lashinger L. M. Weinstock G. M. Bray M. S. (2021). Circadian rhythms and the gut microbiome synchronize the host’s metabolic response to diet. Cell Metab. 33, 873–887. doi: 10.1016/j.cmet.2021.03.015, PMID: 33789092

[B19] He S. Wang J. Zhang J. Xu J. (2021). Intermittent versus continuous energy restriction for weight loss and metabolic improvement: A meta-analysis and systematic review. Obes. (Silver Spring) 29, 108–115. doi: 10.1002/oby.23023, PMID: 34494373

[B20] Heddes M. Altaha B. Niu Y. Reitmeier S. Kleigrewe K. Haller D. . (2022). The intestinal clock drives the microbiome to maintain gastrointestinal homeostasis. Nat. Commun. 13, 6068. doi: 10.1038/s41467-022-33609-x, PMID: 36241650 PMC9568547

[B21] Hou S. Yu J. Li Y. Zhao D. Zhang Z. (2025). Advances in fecal microbiota transplantation for gut dysbiosis-related diseases. Adv. Sci. (Weinh) 12, e2413197. doi: 10.1002/advs.202413197, PMID: 40013938 PMC11967859

[B22] Jakubowicz D. Matz Y. Landau Z. Rosenblum R. C. Twito O. Wainstein J. . (2024). Interaction between early meals (Big-breakfast diet), clock gene mRNA expression, and gut microbiome to regulate weight loss and glucose metabolism in obesity and type 2 diabetes. Int. J. Mol. Sci. 25. doi: 10.3390/ijms252212355, PMID: 39596418 PMC11594859

[B23] Jalanka J. Mattila E. Jouhten H. Hartman J. de Vos W. M. Arkkila P. . (2016). Long-term effects on luminal and mucosal microbiota and commonly acquired taxa in faecal microbiota transplantation for recurrent Clostridium difficile infection. BMC Med. 14, 155. doi: 10.1186/s12916-016-0698-z, PMID: 27724956 PMC5057499

[B24] Jiang Z. D. Jenq R. R. Ajami N. J. Petrosino J. F. Alexander A. A. Ke S. . (2018). Safety and preliminary efficacy of orally administered lyophilized fecal microbiota product compared with frozen product given by enema for recurrent Clostridium difficile infection: A randomized clinical trial. PLoS One 13, e0205064. doi: 10.1371/journal.pone.0205064, PMID: 30388112 PMC6214502

[B25] Khoruts A. Sadowsky M. J. (2016). Understanding the mechanisms of faecal microbiota transplantation. Nat. Rev. Gastroenterol. Hepatol. 13, 508–516. doi: 10.1038/nrgastro.2016.98, PMID: 27329806 PMC5909819

[B26] Kiessling S. Liu S. Haller D. Thaiss C. A. (2025). Origins and functions of microbiome rhythms. Cell Host Microbe 33, 808–819. doi: 10.1016/j.chom.2025.05.017, PMID: 40505620

[B27] Kim H. K. Chijiki H. Nanba T. Ozaki M. Sasaki H. Takahashi M. . (2020). Ingestion of Helianthus tuberosus at Breakfast Rather Than at Dinner Is More Effective for Suppressing Glucose Levels and Improving the Intestinal Microbiota in Older Adults. Nutrients 12. doi: 10.3390/nu12103035, PMID: 33022987 PMC7600786

[B28] Kootte R. S. Levin E. Salojärvi J. Smits L. P. Hartstra A. V. Udayappan S. D. . (2017). Improvement of insulin sensitivity after lean donor feces in metabolic syndrome is driven by baseline intestinal microbiota composition. Cell Metab. 26, 611–619.e6. doi: 10.1016/j.cmet.2017.09.008, PMID: 28978426

[B29] Leone V. Gibbons S. M. Martinez K. Hutchison A. L. Huang E. Y. Cham C. M. . (2015). Effects of diurnal variation of gut microbes and high-fat feeding on host circadian clock function and metabolism. Cell Host Microbe 17, 681–689. doi: 10.1016/j.chom.2015.03.006, PMID: 25891358 PMC4433408

[B30] Leong K. S. W. Jayasinghe T. N. Wilson B. C. Derraik J. G. B. Albert B. B. Chiavaroli V. . (2020). Effects of fecal microbiome transfer in adolescents with obesity: the gut bugs randomized controlled trial. JAMA Netw. Open 3, e2030415. doi: 10.1001/jamanetworkopen.2020.30415, PMID: 33346848 PMC7753902

[B31] Li J. Zhao F. Wang Y. Chen J. Tao J. Tian G. . (2017). Gut microbiota dysbiosis contributes to the development of hypertension. Microbiome 5, 14. doi: 10.1186/s40168-016-0222-x, PMID: 28143587 PMC5286796

[B32] Li T. Rui Z. Mao L. Chang Y. Shao J. Chen Y. . (2023). Eight Weeks of Bifidobacterium lactis BL-99 Supplementation Improves Lipid Metabolism and Sports Performance through Short-Chain Fatty Acids in Cross-Country Skiers: A Preliminary Study. Nutrients 15. doi: 10.3390/nu15214554, PMID: 37960207 PMC10648242

[B33] Li L. Li R. Tian Q. Luo Y. Li R. Lin X. . (2024). Effects of healthy low-carbohydrate diet and time-restricted eating on weight and gut microbiome in adults with overweight or obesity: Feeding RCT. Cell Rep. Med. 5, 101801. doi: 10.1016/j.xcrm.2024.101801, PMID: 39454570 PMC11604488

[B34] Li Y. Zhang S. Li C. Shen J. Cao P. Sun Y. . (2025). Prebiotics chronotherapy alleviates depression-like behaviors in FMT mice through enhancing short-chain fatty acids receptors and intestinal barrier. J. Affect. Disord. 391, 119885. doi: 10.1016/j.jad.2025.119885, PMID: 40664314

[B35] Liang X. Bushman F. D. FitzGerald G. A. (2015). Rhythmicity of the intestinal microbiota is regulated by gender and the host circadian clock. Proc. Natl. Acad. Sci. U.S.A. 112, 10479–10484. doi: 10.1073/pnas.1501305112, PMID: 26240359 PMC4547234

[B36] Litichevskiy L. Thaiss C. A. (2022). The oscillating gut microbiome and its effects on host circadian biology. Annu. Rev. Nutr. 42, 145–164. doi: 10.1146/annurev-nutr-062320-111321, PMID: 35576592

[B37] Lotti S. Dinu M. Colombini B. Amedei A. Sofi F. (2023). Circadian rhythms, gut microbiota, and diet: Possible implications for health. Nutr. Metab. Cardiovasc. Dis. 33, 1490–1500. doi: 10.1016/j.numecd.2023.05.009, PMID: 37246076

[B38] Ma J. Li C. Wang J. Gu J. (2020). Genetically engineered escherichia coli nissle 1917 secreting GLP-1 analog exhibits potential antiobesity effect in high-fat diet-induced obesity mice. Obes. (Silver Spring) 28, 315–322. doi: 10.1002/oby.22700, PMID: 31970910

[B39] Ma F. Li Z. Liu H. Chen S. Zheng S. Zhu J. . (2024). Dietary-timing-induced gut microbiota diurnal oscillations modulate inflammatory rhythms in rheumatoid arthritis. Cell Metab. 36, 2367–2382.e5. doi: 10.1016/j.cmet.2024.08.007, PMID: 39260371

[B40] Min B. H. Devi S. Kwon G. H. Gupta H. Jeong J. J. Sharma S. P. . (2024). Gut microbiota-derived indole compounds attenuate metabolic dysfunction-associated steatotic liver disease by improving fat metabolism and inflammation. Gut Microbes 16, 2307568. doi: 10.1080/19490976.2024.2307568, PMID: 38299316 PMC10841017

[B41] Mocanu V. Zhang Z. Deehan E. C. Kao D. H. Hotte N. Karmali S. (2021). Fecal microbial transplantation and fiber supplementation in patients with severe obesity and metabolic syndrome: a randomized double-blind, placebo-controlled phase 2 trial. Nat. Med. 27, 1272–1279. doi: 10.1038/s41591-021-01399-2, PMID: 34226737

[B42] NCD-RisC N. R. F. C. (2024). Worldwide trends in underweight and obesity from 1990 to 2022: a pooled analysis of 3663 population-representative studies with 222 million children, adolescents, and adults. Lancet 403, 1027–1050. doi: 10.1016/S0140-6736(23)02750-2, PMID: 38432237 PMC7615769

[B43] Ng S. C. Xu Z. Mak J. W. Y. Yang K. Liu Q. Zuo T. . (2022). Microbiota engraftment after faecal microbiota transplantation in obese subjects with type 2 diabetes: a 24-week, double-blind, randomised controlled trial. Gut 71, 716–723. doi: 10.1136/gutjnl-2020-323617, PMID: 33785557

[B44] Niu Y. Heddes M. Altaha B. Birkner M. Kleigrewe K. Meng C. . (2024). Targeting the intestinal circadian clock by meal timing ameliorates gastrointestinal inflammation. Cell Mol. Immunol. 21, 842–855. doi: 10.1038/s41423-024-01189-z, PMID: 38918576 PMC11291886

[B45] Okunogbe A. Nugent R. Spencer G. Powis J. Ralston J. Wilding J. . (2022). Economic impacts of overweight and obesity: current and future estimates for 161 countries. BMJ Glob Health 7. doi: 10.1136/bmjgh-2022-009773, PMID: 36130777 PMC9494015

[B46] Parkar S. G. Kalsbeek A. Cheeseman J. F. (2019). Potential role for the gut microbiota in modulating host circadian rhythms and metabolic health. Microorganisms 7. doi: 10.3390/microorganisms7020041, PMID: 30709031 PMC6406615

[B47] Patikorn C. Roubal K. Veettil S. K. Chandran V. Pham T. Lee Y. Y. . (2021). Intermittent fasting and obesity-related health outcomes: an umbrella review of meta-analyses of randomized clinical trials. JAMA Netw. Open 4, e2139558. doi: 10.1001/jamanetworkopen.2021.39558, PMID: 34919135 PMC8683964

[B48] Reitmeier S. Kiessling S. Clavel T. List M. Almeida E. L. Ghosh T. S. . (2020). Arrhythmic gut microbiome signatures predict risk of type 2 diabetes. Cell Host Microbe 28, 258–272.e6. doi: 10.1016/j.chom.2020.06.004, PMID: 32619440

[B49] Rinott E. Youngster I. Yaskolka Meir A. Tsaban G. Zelicha H. Kaplan A. . (2021). Effects of diet-modulated autologous fecal microbiota transplantation on weight regain. Gastroenterology 160, 158–173.e10. doi: 10.1053/j.gastro.2020.08.041, PMID: 32860791 PMC7755729

[B50] Ross F. C. Patangia D. Grimaud G. Lavelle A. Dempsey E. M. Ross R. P. . (2024). The interplay between diet and the gut microbiome: implications for health and disease. Nat. Rev. Microbiol. 22, 671–686. doi: 10.1038/s41579-024-01068-4, PMID: 39009882

[B51] Ryan P. M. Patterson E. Kent R. M. Stack H. O'Connor P. M. Murphy K. . (2017). Recombinant incretin-secreting microbe improves metabolic dysfunction in high-fat diet fed rodents. Sci. Rep. 7, 13523. doi: 10.1038/s41598-017-14010-x, PMID: 29051554 PMC5648875

[B52] Sasaki H. Miyakawa H. Watanabe A. Nakayama Y. Lyu Y. Hama K. . (2019). Mice microbiota composition changes by inulin feeding with a long fasting period under a two-meals-per-day schedule. Nutrients 11. doi: 10.3390/nu11112802, PMID: 31744168 PMC6893728

[B53] Segers A. Desmet L. Thijs T. Verbeke K. Tack J. Depoortere I. (2019). The circadian clock regulates the diurnal levels of microbial short-chain fatty acids and their rhythmic effects on colon contractility in mice. Acta Physiol. (Oxf) 225, e13193. doi: 10.1111/apha.13193, PMID: 30269420

[B54] Seguella L. Corpetti C. Lu J. Pesce M. Franzin S. B. Palenca I. . (2025). Oleoylethanolamide-producing Lactobacillus paracasei F19 improves metabolic and behavioral disorders by restoring intestinal permeability and microbiota-gut-brain axis in high-fat diet-induced obese male mice. Brain Behav. Immun. 127, 25–44. doi: 10.1016/j.bbi.2025.02.014, PMID: 39988008

[B55] Shen H. Aggarwal N. Cui B. Foo G. W. He Y. Srivastava S. K. . (2025). Engineered commensals for targeted nose-to-brain drug delivery. Cell 188, 1545–1562.e16. doi: 10.1016/j.cell.2025.01.017, PMID: 39914382

[B56] Sudha M. R. Ahire J. J. Jayanthi N. Tripathi A. Nanal S. (2019). Effect of multi-strain probiotic (UB0316) in weight management in overweight/obese adults: a 12-week double blind, randomised, placebo-controlled study. Benef Microbes 10, 855–866. doi: 10.3920/BM2019.0052, PMID: 31965834

[B57] Takahashi J. S. (2017). Transcriptional architecture of the mammalian circadian clock. Nat. Rev. Genet. 18, 164–179. doi: 10.1038/nrg.2016.150, PMID: 27990019 PMC5501165

[B58] Thaiss C. A. Zeevi D. Levy M. Zilberman-Schapira G. Suez J. Tengeler A. C. . (2014). Transkingdom control of microbiota diurnal oscillations promotes metabolic homeostasis. Cell 159, 514–529. doi: 10.1016/j.cell.2014.09.048, PMID: 25417104

[B59] Tompkins T. A. Mainville I. Arcand Y. (2011). The impact of meals on a probiotic during transit through a model of the human upper gastrointestinal tract. Benef Microbes 2, 295–303. doi: 10.3920/BM2011.0022, PMID: 22146689

[B60] Vallianou N. Stratigou T. Christodoulatos G. S. Dalamaga M. (2019). Understanding the role of the gut microbiome and microbial metabolites in obesity and obesity-associated metabolic disorders: current evidence and perspectives. Curr. Obes. Rep. 8, 317–332. doi: 10.1007/s13679-019-00352-2, PMID: 31175629

[B61] Wang L. Ren B. Hui Y. Chu C. Zhao Z. Zhang Y. . (2020). Methionine restriction regulates cognitive function in high-fat diet-fed mice: roles of diurnal rhythms of SCFAs producing- and inflammation-related microbes. Mol. Nutr. Food Res. 64, e2000190. doi: 10.1002/mnfr.202000190, PMID: 32729963

[B62] Wang J. Wu P. Chen X. D. Yu A. Dhital S. (2025). Effect of food matrix and administration timing on the survival of lactobacillus rhamnosus GG during *in vitro* gastrointestinal digestion. Foods (Basel Switzerland) 14, 3076. doi: 10.3390/foods14173076, PMID: 40941193 PMC12428712

[B63] Wang P. Wang R. Zhao W. Zhao Y. Wang D. Zhao S. . (2025). Gut microbiota-derived 4-hydroxyphenylacetic acid from resveratrol supplementation prevents obesity through SIRT1 signaling activation. Gut Microbes 17, 2446391. doi: 10.1080/19490976.2024.2446391, PMID: 39725607 PMC12931687

[B64] Wilson B. C. Vatanen T. Jayasinghe T. N. Leong K. S. W. Derraik J. G. B. Albert B. B. . (2021). Strain engraftment competition and functional augmentation in a multi-donor fecal microbiota transplantation trial for obesity. Microbiome 9, 107. doi: 10.1186/s40168-021-01060-7, PMID: 33985595 PMC8120839

[B65] Woo S. G. Kim S. K. Lee S. G. Lee D. H. (2025). Engineering probiotic Escherichia coli for inflammation-responsive indoleacetic acid production using RiboJ-enhanced genetic circuits. J. Biol. Eng. 19, 10. doi: 10.1186/s13036-025-00479-y, PMID: 39838372 PMC11753152

[B66] Xia J. Guo W. Hu M. Jin X. Zhang S. Liu B. . (2023). Resynchronized rhythmic oscillations of gut microbiota drive time-restricted feeding induced nonalcoholic steatohepatitis alleviation. Gut Microbes 15, 2221450. doi: 10.1080/19490976.2023.2221450, PMID: 37309179 PMC10266122

[B67] Xu H. Fang F. Wu K. Song J. Li Y. Lu X. . (2023). Gut microbiota-bile acid crosstalk regulates murine lipid metabolism via the intestinal FXR-FGF19 axis in diet-induced humanized dyslipidemia. Microbiome 11, 262. doi: 10.1186/s40168-023-01709-5, PMID: 38001551 PMC10675972

[B68] Ye Y. Xu H. Xie Z. Wang L. Sun Y. Yang H. . (2020). Time-restricted feeding reduces the detrimental effects of a high-fat diet, possibly by modulating the circadian rhythm of hepatic lipid metabolism and gut microbiota. Front. Nutr. 7, 596285. doi: 10.3389/fnut.2020.596285, PMID: 33425971 PMC7793950

[B69] Yin J. Li Y. Han H. Ma J. Liu G. Wu X. . (2020). Administration of exogenous melatonin improves the diurnal rhythms of the gut microbiota in mice fed a high-fat diet. mSystems 5. doi: 10.1128/msystems.00002-20, PMID: 32430404 PMC7253360

[B70] Yu E. W. Gao L. Stastka P. Cheney M. C. Mahabamunuge J. Torres Soto M. . (2020). Fecal microbiota transplantation for the improvement of metabolism in obesity: The FMT-TRIM double-blind placebo-controlled pilot trial. PLoS Med. 17, e1003051. doi: 10.1371/journal.pmed.1003051, PMID: 32150549 PMC7062239

[B71] Zarrinpar A. Chaix A. Yooseph S. Panda S. (2014). Diet and feeding pattern affect the diurnal dynamics of the gut microbiome. Cell Metab. 20, 1006–1017. doi: 10.1016/j.cmet.2014.11.008, PMID: 25470548 PMC4255146

[B72] Zecheng L. Donghai L. Runchuan G. Yuan Q. Qi J. Yijia Z. . (2023). Fecal microbiota transplantation in obesity metabolism: A meta analysis and systematic review. Diabetes Res. Clin. Pract. 202, 110803. doi: 10.1016/j.diabres.2023.110803, PMID: 37356723

[B73] Zhang Z. Mocanu V. Deehan E. C. Hotte N. Zhu Y. Wei S. . (2024). Recipient microbiome-related features predicting metabolic improvement following fecal microbiota transplantation in adults with severe obesity and metabolic syndrome: a secondary analysis of a phase 2 clinical trial. Gut Microbes 16, 2345134. doi: 10.1080/19490976.2024.2345134, PMID: 38685731 PMC11062372

[B74] Zhao C. Lei S. Zhao H. Li Z. Miao Y. Peng C. . (2025). Theabrownin remodels the circadian rhythm disorder of intestinal microbiota induced by a high-fat diet to alleviate obesity in mice. Food Funct. 16, 1310–1329. doi: 10.1039/D4FO05947F, PMID: 39866149

